# From Lipid Composition to Electroporation: Mechanical Encoding of Barrier Resilience in Stratum Corneum Membranes

**DOI:** 10.1021/acsomega.6c04148

**Published:** 2026-09-01

**Authors:** Neila Cristina Fonseca Machado, Nicolás A. García, Nuria Anguita-Ortiz, Lorena Ruano, Juan J. Nogueira, Herculano da Silva Martinho

**Affiliations:** † Universidade Federal do ABC, Centro de Ciências Naturais e Humanas, Av. dos Estados 5001, Santo André, São Paulo 09210-580, Brasil; ‡ 28249 Universidad Nacional del Sur, Av.L.N.Alem, 1253, Bahía Blanca, Buenos Aires B8000CPB, Argentina; § Universidad Autónoma de Madrid, Chemistry Department and IADCHEM, Institute for Advanced Research in Chemistry, Universidad Autónoma de Madrid, Calle Francisco Tomás y Valiente, 7, Madrid 28049, Spain

## Abstract

The permeability of the skin barrier is not fixed—it emerges from the molecular organization of its lipid matrix. In the stratum corneum, compositional alterations associated with dermatological conditions reshape this organization, yet how these changes influence electrically induced transport remains unclear. Here, we employ fully atomistic nonequilibrium molecular dynamics simulations to uncover how lipid composition is mechanically encoded into electroporation response in biomimetic SC lipid membranes. Eleven membrane models spanning ceramide-rich, cholesterol-rich, and physiologically balanced compositions were systematically investigated. We find that electroporation susceptibility is not dictated by individual lipid species but by the collective mechanical state encoded in membrane packing. Cholesterol-rich membranes exhibit rapid pore nucleation and form large conductive defects, whereas ceramide-dominated systems resist pore formation within the simulated time scale. Intermediate compositions display delayed nucleation and reduced pore sizes. The physiologically balanced SCbase membrane exhibits delayed pore formation and comparatively small conductive defects, consistent with a mechanically resilient yet not pore-immune regime. By linking pore nucleation events and time-resolved water transport to equilibrium structural descriptors, we show that bilayer thickness, interfacial cohesion, and lipid organization collectively define the effective barrier to defect formation. These results establish a predictive framework connecting compositional alterations—relevant to both healthy and compromised skin—to electrically induced permeability, providing molecular-level insights into barrier resilience and strategies for controlled transdermal transport.

## Graphical abstract

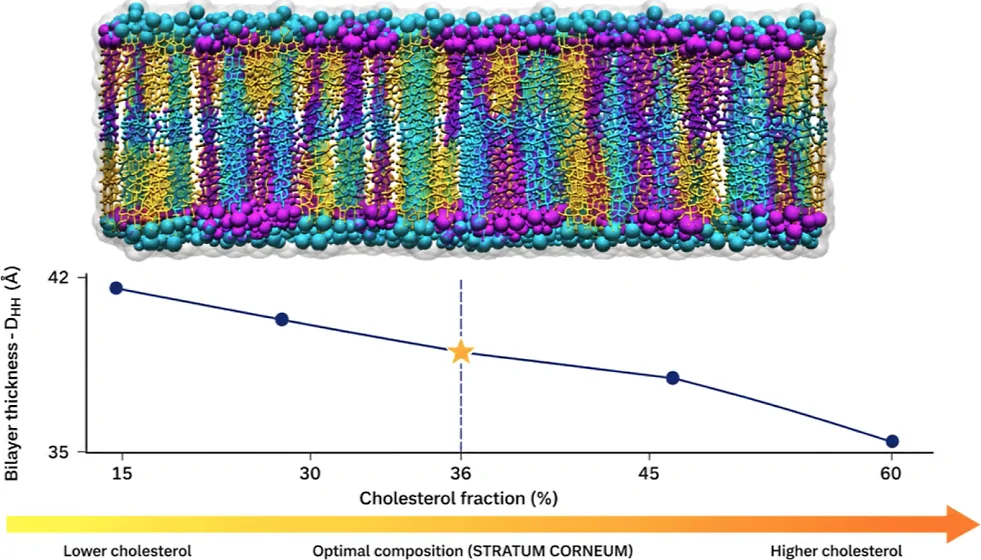

## Introduction

The stratum corneum (SC) constitutes the primary permeability barrier of human skin, regulating transepidermal water loss and protecting the organism against chemical and physical insults. Its barrier function arises from a highly organized intercellular lipid matrix composed predominantly of ceramides (CER), cholesterol (CHL), and free fatty acids (FA) arranged in lamellar phases.^1–4^ In contrast to phospholipid bilayers, SC membranes exhibit strong interfacial cohesion, low hydration, and restricted molecular mobility, which collectively suppress passive molecular transport.^5,6^

While this exceptional barrier is essential for physiological protection, it also limits the transdermal delivery of therapeutic and cosmetic compounds. Strategies capable of transiently and controllably modulating SC permeability are therefore of considerable interest. Among these, externally applied electric fields have emerged as an effective approach to enhance transport without permanently disrupting tissue integrity.^7–9^

Techniques such as iontophoresis and electroporation promote molecular transport across the SC by inducing nanoscale aqueous defects and driving charged species through the lipid matrix.^7,8,10–12^ Beyond their practical relevance, electric fields provide a controlled physical perturbation to probe barrier stability. In phospholipid bilayers, electroporation has been extensively characterized as a field-driven process involving membrane polarization, defect nucleation, and stabilization of water-filled conductive pathways.^13–18^

However, SC lipid assemblies differ fundamentally from phospholipid membranes in thickness, compositional heterogeneity, and interfacial organization.^3,4^ These structural characteristics are directly linked to physiological function and to the response under external perturbations. In particular, lipid composition plays a central role in regulating barrier integrity and permeability.^1,19,20^

Such compositional variations are strongly associated with barrier dysfunction in inflammatory skin disorders. In conditions such as atopic dermatitis, reductions in ceramide content and changes in the CER/CHL ratio have been linked to increased transepidermal water loss and enhanced permeability.^19,21–23^ These alterations disrupt lamellar organization and weaken interfacial cohesion, ultimately compromising barrier integrity.^20^

From a molecular perspective, these compositional changes can be interpreted as perturbations of the intrinsic mechanical landscape of the SC lipid matrix. Variations in lipid composition reshape both equilibrium structure and the response to external perturbations.

Within this framework, electroporation corresponds to the formation of transient aqueous defects within the membrane under an external electric field. The applied field promotes membrane polarization and facilitates water penetration and stabilization within the lipid interior.^13,15^ As a result, pore formation emerges as a barrier-crossing event whose probability depends sensitively on the underlying structural organization and follows a nucleation-driven, inherently stochastic process.^17,24,25^

In SC lipid assemblies, organization arises from a balance between interfacial cohesion, vertical packing, and lateral lipid arrangement. These features collectively define the intrinsic mechanical state of the system and govern its response to external perturbations. Establishing how this equilibrium organization encodes electroporation susceptibility—and, in particular, how it controls the statistics of pore nucleation events—remains an open challenge for compositionally complex SC biomimetic membranes.

In this context, the set of membrane compositions investigated in this work spans a broad compositional space encompassing both idealized and physiologically relevant conditions, including ceramide-rich, cholesterol-rich, mixed systems, and a composition representative of healthy SC. Rather than treating these systems as independent cases, this compositional space provides a controlled framework to probe how lipid balance encodes stability and susceptibility to electric-field-induced pore formation.

To address this challenge, molecular dynamics simulations provide a powerful framework. Under sufficiently strong constant electric fields, pore formation can be directly induced in silico, enabling atomistic investigation of electroporation-like behavior. Moreover, by enabling systematic compositional variation and direct access to structural descriptors such as bilayer thickness (*D*_HH_), chain order parameters (*S*_CD_), lipid tilt, and area per lipid (APL), simulations allow the intrinsic mechanical state to be quantified at molecular resolution.^13,26–28^ These descriptors collectively define the mechanical landscape governing defect nucleation under external perturbation.

Here, we employ fully atomistic nonequilibrium molecular dynamics simulations to investigate how lipid composition controls electric-field-induced pore formation in biomimetic SC membranes. By systematically varying ceramide, cholesterol, and fatty-acid content across 11 compositions, we construct a compositional landscape spanning both idealized and physiologically relevant membrane states.

By correlating equilibrium structural organization with pore formation behavior under a constant electric field, we establish a direct link between lipid composition, membrane mechanics, and electroporation susceptibility. This approach provides a physically grounded and predictive framework to rationalize how compositional variations—relevant to both healthy and compromised skin—govern barrier response under electrical perturbation.

## Methodology

### Membrane Compositions

A set of 11 lipid membranes was constructed to systematically probe compositional effects on structural organization and electroporation response. The systems include single-component, binary, and ternary mixtures spanning a broad compositional range. The models are identified using a numerical labeling scheme, while their detailed compositions are summarized in Table 1.

**Table 1:** Compositional Landscape of the Biomimetic SC Membrane Models Investigated in This Work^a^

model	cholesterol (CHL) %	fatty acid (FA) %	ceramide (CER) %	*N*_rep_
model 1 (CER100)	0	0	100	3
model 2 (CHL100)	100	0	0	3
model 3 (FA100)	0	100	0	3
model 4 (FA50/CER50)	0	50	50	3
model 5 (FA50/CHL50)	50	50	0	3
model 6 (CHL50/CER50)	50	0	50	3
model 7	15	39	46	5
model 8	30	39	31	5
model 9	45	39	16	5
model 10	60	39	1	5
model 11 (SCbase)	36	39	25	5

a *N*_rep_ denotes the number of independent replicas simulated for each composition. Pore nucleation analyses were performed considering all replicas, with classification into *no wetting* and *sustained pore* as described below.

Models 7–10 define a controlled compositional pathway in which the cholesterol fraction increases monotonically while the fatty-acid content remains fixed, enabling isolation of cholesterol-dependent structural reorganization effects.

Model 11 corresponds to a physiologically relevant reference composition (SCbase), which serves as a structural and mechanical benchmark.

All membranes were assembled as symmetric bilayers with fully hydrated lamellar organization, enabling systematic investigation of compositional effects on membrane structure and electroporation response.

### Molecular Dynamics Simulations

All molecular dynamics simulations were performed using AMBER22 ^29^ with the Lipid21 force field.^30^ The models were constructed using the CHARMM-GUI Membrane Builder.^31^ Systems were solvated in explicit TIP3P water,^32^ with Na^+^ and Cl^–^ ions added to ensure electroneutrality.

Energy minimization consisted of 10,000 steps (5000 steepest descent followed by 5000 conjugate gradient). Equilibration proceeded in three stages: (i) 125 ps *NVT* with positional restraints on lipid heavy atoms, (ii) 125 ps *NVT* without restraints, and (iii) 125 ps semi-isotropic NPT at 1 bar using a Monte Carlo barostat.

Temperature was maintained at 303.15 K using a Langevin thermostat (collision frequency 1 ps^–1^). A 1 fs time step was used during equilibration. SHAKE^33^ constraints were applied to bonds involving hydrogen atoms, and particle mesh Ewald^34^ electrostatics were computed with a 12 Å cutoff.

### Field-Free Simulations

Production simulations without an electric field were carried out for 200 ns in the *NPT* ensemble using a 2 fs time step. The simulation conditions were identical to those used during equilibration. These trajectories define the equilibrium structural baseline of each membrane.

### Simulations under Electric Field

A constant electric field was applied along the membrane normal (*z* direction), introducing an additional force on each charged particle

$$F_{i}=q_{i}E$$

where *q*_*i*_ is the charge of particle *i* and **E** is the applied field. This formulation corresponds to the addition of a linear electrostatic potential across the simulation box, effectively generating a transmembrane voltage and inducing membrane polarization.^13–15^

Under sufficiently strong constant electric fields, lipid membranes undergo electric-field-induced pore formation, enabling the investigation of electroporation-like nucleation and growth processes at atomistic resolution. In this work, the applied field is used as a controlled perturbation to probe the susceptibility of different membrane compositions to pore formation under identical electrical conditions.

The field was applied using the efz parameter in AMBER, with box scaling disabled (efn = 0). Simulations were performed for up to 1 μs or until pore formation or complete membrane disruption was observed. Pore formation was defined as the emergence of a persistent aqueous pathway across the membrane, as quantified by the hydration-based criterion described below.

Multiple independent replicas were performed for each model (Table 1) to account for the inherent variability of pore nucleation.

The instantaneous temperature was monitored throughout the electric-field simulations to verify that the applied field did not introduce artificial thermal effects. Representative temperature profiles are provided in Figure S4 of the Supporting Information.

### Selection of Electric Field Intensity

The electric-field intensity was selected to induce pore nucleation within accessible simulation time scales while preserving membrane integrity. In atomistic simulations, relatively high field strengths are typically required to observe barrier-crossing events within feasible simulation times.^13,15^

The field magnitude was calibrated using the SCbase membrane as a reference system. In previous coarse-grained Martini simulations,^10^ pore nucleation occurred at *E*_*z*_ = 0.0228 V nm^–1^. Due to differences in electrostatic representation between coarse-grained and atomistic models, the corresponding field strength was determined independently within the Lipid21 framework.

A systematic screening was conducted over the range 0.525–2.400 V nm^–1^. No pore nucleation was observed for *E*_*z*_ ≤ 1.50 V nm^–1^ within the simulated time scale, whereas fields between 1.60 and 1.70 V nm^–1^ produced only delayed nucleation events (>100 ns). In contrast, fields between 1.71 and 1.80 V nm^–1^ reproducibly induced electric-field-driven pore formation within accessible simulation times while preserving membrane integrity.

At *E*_*z*_ = 1.739 V nm^–1^, the SCbase membrane exhibited a reproducible deformation characterized by prenucleation curvature along the field direction, followed by the formation of a hydrated defect spanning the membrane interior. This behavior reflects polarization-driven bending that facilitates defect nucleation. Based on these observations, *E*_*z*_ = 1.739 V nm^–1^ was selected as the reference field for all compositional comparisons.

Field strengths above ∼2.40 V nm^–1^ led to rapid membrane disintegration and were therefore excluded from analysis.

### Structural Analysis

Structural descriptors were used to quantify membrane organization and relate equilibrium structure to pore nucleation behavior. For porated trajectories, observables were evaluated relative to the pore-opening time, while for nonporating systems, analysis was restricted to the final 100 ns.

Bilayer thickness *D*_HH_ was obtained from headgroup density profiles along the membrane normal. Area per lipid (APL) was computed from the lateral box dimensions divided by the number of lipids per leaflet.

Chain ordering was quantified using deuterium order parameters *S*_CD_, and cholesterol orientation was characterized using the C3–C17 sterol axis (*S*_axis_^CHL^). Tilt angles were calculated relative to the membrane normal for all lipid species.

### Pore Detection and Size Estimation

Pore nucleation was treated as a barrier-crossing event governed by thermal fluctuations, corresponding to the formation of a persistent hydrated defect spanning the membrane interior.

Pore formation was identified by monitoring water occupancy within a central membrane slab defined relative to the instantaneous lipid center of mass. A pore-opening time (*t*_pore_) was assigned when the number of water molecules exceeded 20 for at least five consecutive analyzed frames (analysis interval 0.1 ns).

Each trajectory was classified as either *no wetting* or *sustained pore*.

For *sustained pore* trajectories, pore size was estimated by projecting water molecules within a 1.0 nm slab onto the membrane plane and discretizing them onto a two-dimensional grid (0.3 nm resolution). The largest connected cluster was identified and converted into an effective circular radius. Maximum (*r*_max_) and final (*r*_final_) radii were extracted within a 5 ns window after pore nucleation.

This procedure was applied consistently to all porating trajectories, enabling direct quantitative comparison of pore size across compositions and independent replicas.

This combined detection and characterization protocol enables consistent identification of pore nucleation events and quantitative comparison of their structural signatures across different membrane compositions.

### Water-Crossing Flux

To further characterize the progressive loss of membrane integrity under electrical loading, an area-normalized early water-crossing flux was quantified from the onset of the electric field until 5 ns after pore nucleation. Water molecules were tracked relative to the instantaneous membrane center of mass and classified as belonging to the upper aqueous compartment, membrane core, or lower aqueous compartment. A transmembrane crossing event was recorded only when a water molecule persistently traversed the membrane from one bulk aqueous compartment to the opposite one after crossing the membrane core, using a persistence criterion of five consecutive analyzed frames to eliminate transient fluctuations. The cumulative number of crossing events was subsequently normalized by the mean projected membrane area and by the corresponding analysis time (0 → *t*_pore_ + 5 ns), yielding an area-normalized early water-crossing flux (molecules nm^–2^ ns^–1^). This metric provides a dynamic descriptor of the progressive loss of membrane integrity during the early stages of electroporation and enables direct comparison among membrane compositions exhibiting different pore nucleation times.

A representative time-resolved example of this analysis is provided in Figure S3, and the per-replica values of *J*_water_ are reported in Table S2.

## Results and Discussion

### Control Membranes Define a Compositional Mechanical Baseline

To establish the intrinsic mechanical state prior to electrical perturbation, we quantified a set of equilibrium structural descriptors for all control membranes, including bilayer thickness (*D*_HH_), chain order parameters, orientational tilt, and lateral packing (APL). The complete data set, including lipid-specific order and tilt metrics, is provided in Table S1 (Supporting Information).

### Interfacial Stratification, Headgroup Architecture, and Latent Permeability

The equilibrium structural organization of all control membranes is summarized in the 3 × 3 structural panel (Figure S1, Supporting Information). The top row (total density) defines the global bilayer envelope and thickness, the middle row (headgroup densities) resolves interfacial stratification, and the bottom row (water density) reports on core hydration and latent permeability.

Across all compositions, a consistent biomimetic SC lipid membrane architecture is preserved. Ceramides define interfacial slabs, fatty acids populate the bilayer midplane, and cholesterol occupies an intermediate vertical position. Compositional variation does not disrupt lamellar organization but modulates its sharpness, thickness, and hydration state.

Two equilibrium signatures emerge as key indicators of intrinsic barrier robustness: (i) the sharpness and symmetry of the interfacial headgroup peaks (Figure S1D–F), reflecting interfacial cohesion and packing regularity, and (ii) the presence or absence of measurable water density at the bilayer center (Figure S1G–I), which is indicative of latent permeability.

CER100 (model 1) defines the cohesive extreme: sharp and symmetric headgroup peaks, a well-defined total density envelope, and complete core dehydration indicate strong ceramide-mediated lamellar locking. In contrast, CHL100 (model 2) forms the thinnest bilayer and exhibits broadened headgroup distributions, reflecting enhanced orientational freedom and vertical reorganization. Although macroscopically stable in the absence of a field, this state is consistent with a mechanically permissive architecture.

FA100 (model 3) presents a distinct behavior. Despite its large apparent thickness in the total density profile, the headgroup organization is less cohesive, and the water density profile reveals abnormal core hydration already under field-free conditions. This intrinsic instability complicates a clear separation between equilibrium fragility and field-induced poration; model 3 is therefore not included in the quantitative electroporation analysis.

Taken together, the 3 × 3 panel reveals that compositional tuning primarily modifies interfacial cohesion, vertical organization, and latent hydration without destroying lamellar order. These equilibrium fingerprints define the mechanical baseline upon which electric-field-induced defect nucleation acts.

### Cholesterol-Dependent Thinning in Models 7–10

Within models 7–10, bilayer thickness decreases monotonically with increasing cholesterol fraction (Figure 1). Importantly, this thinning occurs without substantial variation in APL (Table S1), indicating that cholesterol modulation in biomimetic SC lipid membranes primarily reflects vertical and orientational reorganization rather than large-scale lateral expansion.

**Figure 1:**
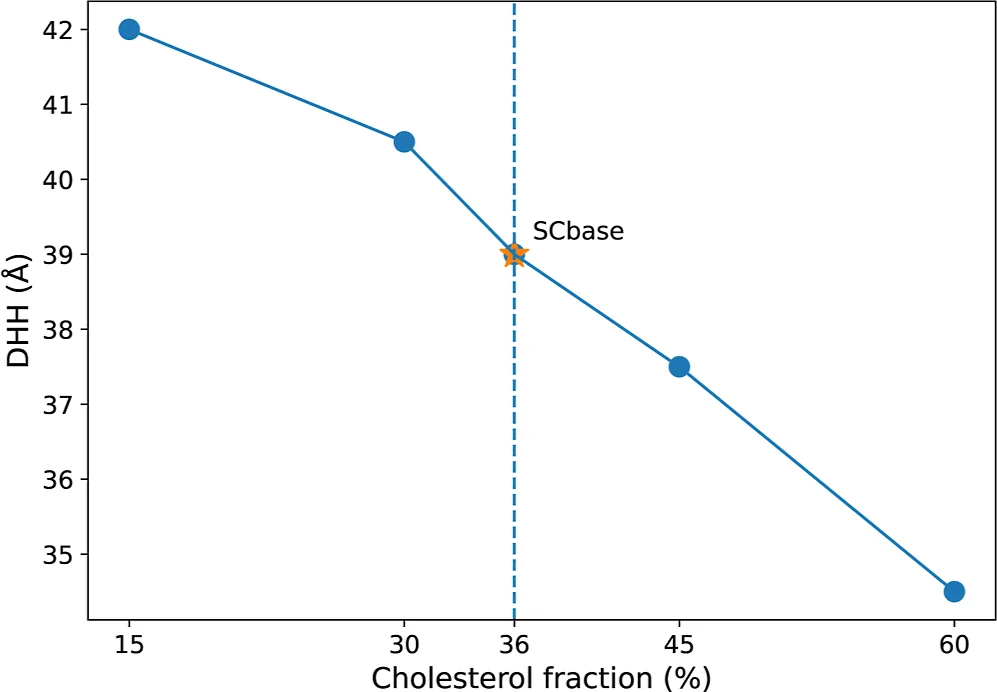
Cholesterol-dependent modulation of bilayer thickness under control conditions. Headgroup-to-headgroup distance (*D*_HH_) as a function of cholesterol fraction for models 7–10 and SCbase (model 11). Markers represent equilibrium values extracted from the final 100 ns of representative field-free trajectories (see Table S1 for full statistics).

The SCbase composition (model 11, 36% CHL) occupies an intermediate position along this structural continuum, defining a balanced packing regime between ceramide-dominated rigidity and cholesterol-driven thinning. As shown in Figure 1, increasing cholesterol fraction compresses the vertical bilayer dimension while preserving overall lateral area, reinforcing the view that electroporation susceptibility is encoded predominantly in vertical packing and interfacial architecture.

Taken together, the structural descriptors summarized in Table S1 define the full equilibrium landscape of biomimetic SC lipid membranes. However, not all observables contribute equally to electroporation susceptibility, motivating a reduced descriptor set.

These reduced descriptors are summarized in Table 2 and define the intrinsic mechanical landscape governing defect nucleation. In this representation, cholesterol-dependent thinning (Figure 1) defines the primary vertical control axis, while ceramide ordering and tilt encode interfacial locking strength. Because APL varies only weakly across models 7–10, susceptibility is primarily modulated by vertical and orientational reorganization rather than global lateral expansion.

**Table 2:** Core Mechanistic Landscape of Biomimetic SC Lipid Membranes under Electric Loading (*E*_*z*_ = 1.739 V nm^–1^)­^a^

model	*D*_HH_ (Å)	*S*_CD_^CER^	Tilt_CER_ (deg)	APL (nm^2^)
model 1 (CER100)	41.5	–0.407	13.9	0.383 ± 0.001
model 2 (CHL100)	33.5	–	–	0.381 ± 0.001
model 4 (FA50/CER50)	43.5	–0.409	14.2	0.286 ± 0.001
model 5 (FA50/CHL50)	41.5	–	–	0.290 ± 0.001
model 6 (CHL50/CER50)	37.0	–0.366	16.7	0.385 ± 0.001
model 7 (CHL15/FA39/CER46)	42.0	–0.402	14.2	0.307 ± 0.001
model 8 (CHL30/FA39/CER31)	40.5	–0.372	16.9	0.308 ± 0.001
model 9 (CHL45/FA39/CER16)	37.5	–0.366	15.8	0.309 ± 0.001
model 10 (CHL60/FA39/CER01)	34.5	–0.371	19.4	0.307 ± 0.001
model 11 (SCbase)	39.0	–0.366	18.0	0.309 ± 0.001

a Structural descriptors were computed from the final 100 ns of field-free simulations and define the intrinsic mechanical baseline governing pore nucleation susceptibility. Replica-to-replica variations are negligible compared to compositional trends.

This mechanistic interpretation is further supported by density-resolved analyses (Figure S1) and the full structural data set reported in Table S1, while pore nucleation events are reported at the per-replica level in Table S2 (Supporting Information).

### Chain Ordering, Tilt, and Lateral Packing

Chain order parameters reinforce the interfacial hierarchy identified in the core mechanistic table (Table 2). Ceramides exhibit the highest orientational order among biomimetic SC lipid membranes, reflecting their role as the primary interfacial scaffold. Across models 7–10, increasing cholesterol fraction induces a modest reduction in $\left|S_{CD}^{CER}\right|$ and a systematic shift in ceramide tilt, indicating enhanced conformational flexibility within a vertically compressed architecture. In contrast, the sterol nucleus remains highly aligned relative to the membrane normal (high *S*_axis_^CHL^; Table S1), demonstrating that cholesterol reorganizes the vertical packing without losing orientational coherence.

Notably, lateral packing remains nearly invariant across models 7–10 (Figure 2), despite pronounced cholesterol-dependent thinning (Figure 1).

**Figure 2:**
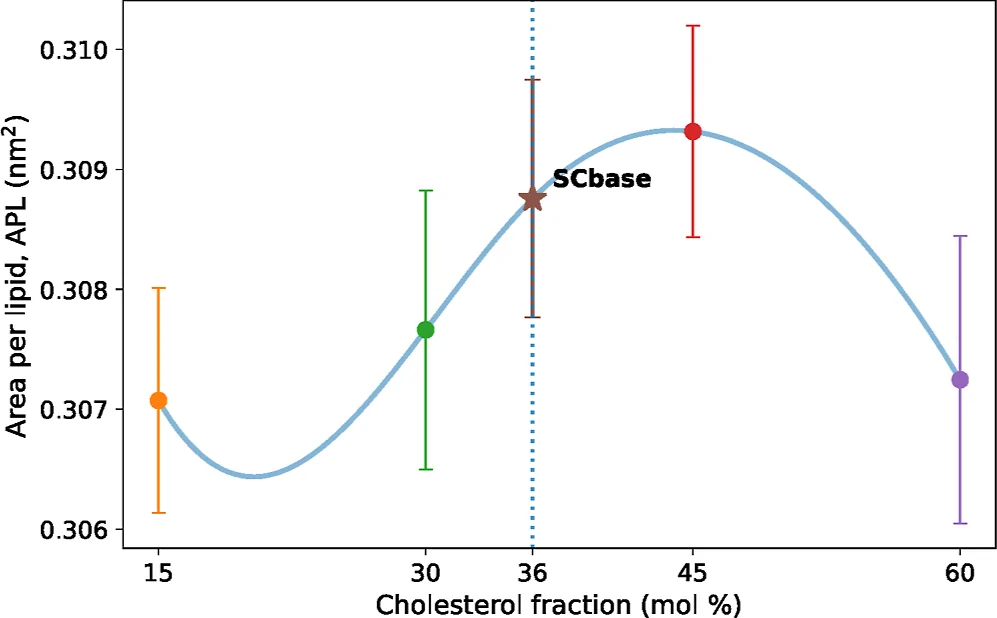
Area per lipid (APL) as a function of cholesterol fraction under control conditions. APL values were computed from field-free trajectories (final 100 ns). Variations across replicas are negligible (Table S1). The weak variation in APL across models 7–10 highlights the preservation of lateral packing despite cholesterol-dependent bilayer thinning. The star highlights the SCbase composition (model 11, 36% CHL), and the vertical dashed line marks the physiological cholesterol fraction.

Taken together, *D*_HH_, *S*_CD_^CER^, Tilt_CER_, and APL form a mechanically self-consistent descriptor set that defines the equilibrium nucleation landscape prior to electrical perturbation.

This decoupling between vertical compression (*D*_HH_) and lateral area (APL) confirms that cholesterol primarily modulates biomimetic SC lipid membranes through vertical and orientational reorganization rather than through substantial lateral packing changes.

Figure 3 provides a direct visualization of the ceramide response along the cholesterol-composition pathway. While the magnitude of *S*_CD_^CER^ decreases from model 7 to model 8 and subsequently reaches a plateau, ceramide tilt exhibits an overall increase with cholesterol enrichment. The SCbase membrane occupies an intermediate regime, combining moderate ceramide ordering with elevated tilt. Together with the cholesterol-dependent thinning shown in Figure 1 and the weak APL variation shown in Figure 2, these observations indicate that cholesterol enrichment primarily promotes vertical and orientational reorganization of the membrane without substantial changes in lateral packing. Together, these descriptors suggest that cholesterol-induced changes in membrane packing architecture establish the structural conditions that precede and modulate pore nucleation under electrical perturbation.

**Figure 3:**
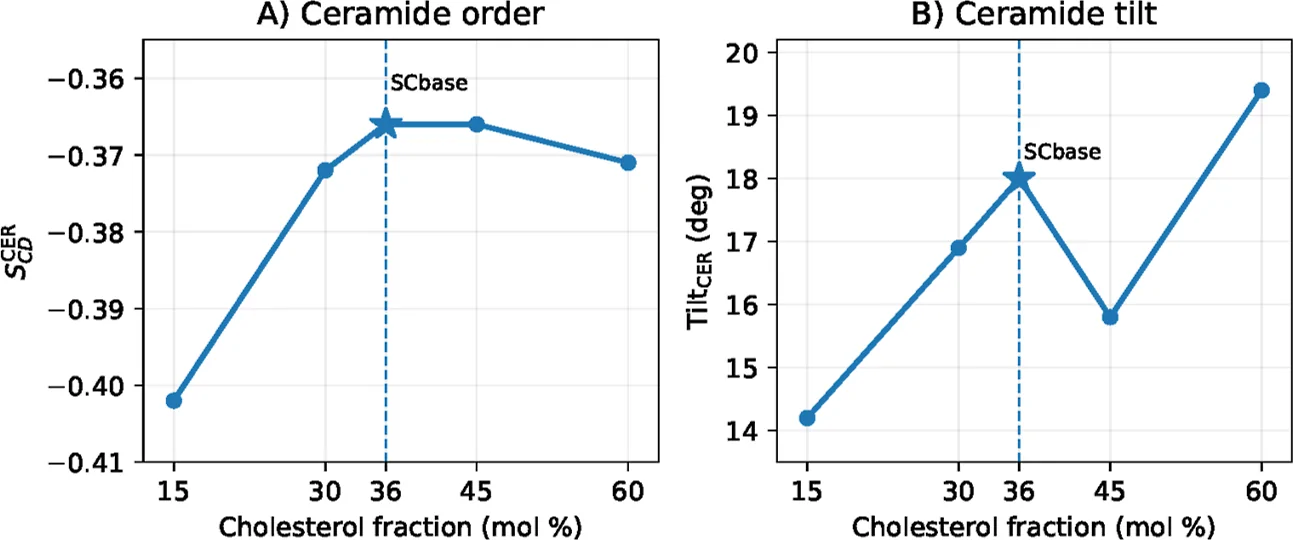
Ceramide ordering and orientational response along the cholesterol-composition pathway. (A) Chain-averaged ceramide deuterium order parameter, *S*_CD_^CER^, and (B) ceramide tilt angle as a function of cholesterol fraction for models 7–10 and SCbase. The SCbase composition is highlighted by a star, and the vertical dashed line indicates the physiological cholesterol fraction (36 mol %). The descriptors were extracted from field-free trajectories using the final 100 ns and provide a direct representation of ceramide ordering and orientational organization along the cholesterol-composition pathway.

All structural descriptors discussed in this work (*D*_HH_, *S*_CD_, tilt, and APL) were obtained from independent field-free equilibrium trajectories and were used to define the intrinsic mechanical baseline of each membrane composition. The subsequent electric-field simulations were intentionally performed under nonequilibrium conditions to probe the susceptibility of these equilibrium membrane states to pore nucleation under a common external perturbation.

To verify that the applied electric field did not introduce artificial thermal effects during the simulations, we monitored the instantaneous temperature throughout representative electric-field trajectories for all membrane compositions. The temperature remained stable around the target value of 300 K throughout the simulations (Figure S4, Supporting Information), with no systematic temperature drift or progressive field-induced heating observed. These observations demonstrate that the Langevin thermostat efficiently maintained thermal regulation throughout the simulations, indicating that the membrane responses discussed below reflect the electromechanical action of the applied electric field rather than artificial heating.

To complement the quantitative water-crossing flux analysis presented in the main text, the Supporting Information provides additional time-resolved characterization of water transport and membrane integrity. Figure S3 illustrates the complete water-integrity analysis workflow for a representative SCbase trajectory, including membrane-core hydration, cumulative transmembrane water crossings, and instantaneous crossing rate, thereby illustrating the complete construction of the water-integrity metric. In addition, Figure S2 presents representative cumulative water-crossing trajectories (seed 1) for all membrane compositions, providing a direct time-resolved comparison of water transport under electrical loading. Per-replica values of the area-normalized early water-crossing flux are reported in Table S2.

Together, these supplementary analyses provide additional methodological transparency while complementing the quantitative comparison presented in the main manuscript with a direct time-resolved visualization of membrane barrier integrity under electrical loading.

### Composition-Dependent Pore Nucleation under Constant Electric Field

We next applied a constant electric field and quantified pore nucleation using a hydration-based persistence criterion. Because pore nucleation is inherently stochastic, observed pore-opening times represent realizations of an underlying distribution rather than deterministic quantities. Accordingly, comparisons are made in terms of relative susceptibility across compositions rather than absolute nucleation times. When pore formation occurred, pore-size descriptors (*r*_max_ and *r*_final_) were extracted from the dominant water-filled cluster. Each replica was classified as either *no wetting* (no persistent hydration event within the simulated time window) or *sustained pore* (a pore-opening event detected by the slab-water persistence criterion). Per-seed results are listed in Table S2, and per-model statistics (mean ± standard deviation over *sustained pore* replicas) are summarized in Table 3.

**Table 3:** Pore Nucleation Statistics under a Constant Electric Field (*E*_*z*_ = 1.739 V nm^–1^)­^a^

model	*N*	*N*_sus_	*t*_pore_ (ns)	*r*_max_ (nm)
model 1 (CER100)	3	0	–	–
model 2 (CHL100)	3	3	5.37 ± 1.99	2.92 ± 0.39
model 4 (FA50/CER50)	3	0	–	–
model 5 (FA50/CHL50)	3	3	23.20 ± 22.06	0.797 ± 0.150
model 6 (CHL50/CER50)	3	3	49.23 ± 40.78	2.831 ± 0.315
model 7 (CHL15/FA39/CER46)	5	5	20.92 ± 7.69	1.35 ± 0.18
model 8 (CHL30/FA39/CER31)	5	5	13.10 ± 6.54	0.78 ± 0.18
model 9 (CHL45/FA39/CER16)	5	5	18.74 ± 3.74	1.60 ± 0.22
model 10 (CHL60/FA39/CER01)	5	0	–	–
model 11 (SCbase)	5	5	289.08 ± 181.16	0.904 ± 0.283

a *N* denotes the number of independent replicas and *N*_sus_ the number of trajectories classified as *sustained pore*. Pore-opening times (*t*_pore_) and maximum pore radii (*r*_max_) are reported as mean ± standard deviation over *sustained pore* trajectories. Systems with no pore nucleation are classified as *no wetting*.

### Pore-Opening Statistics at *E*_*z*_ = 1.739 V nm^–1^

Pore nucleation is strongly composition-dependent (Table 3 and Figure 4), reflecting the intrinsic mechanical landscape defined under control conditions. Model 1 (CER100) showed no pore nucleation events across all replicas within the simulated time window, consistent with a highly cohesive, tightly packed barrier.

**Figure 4:**
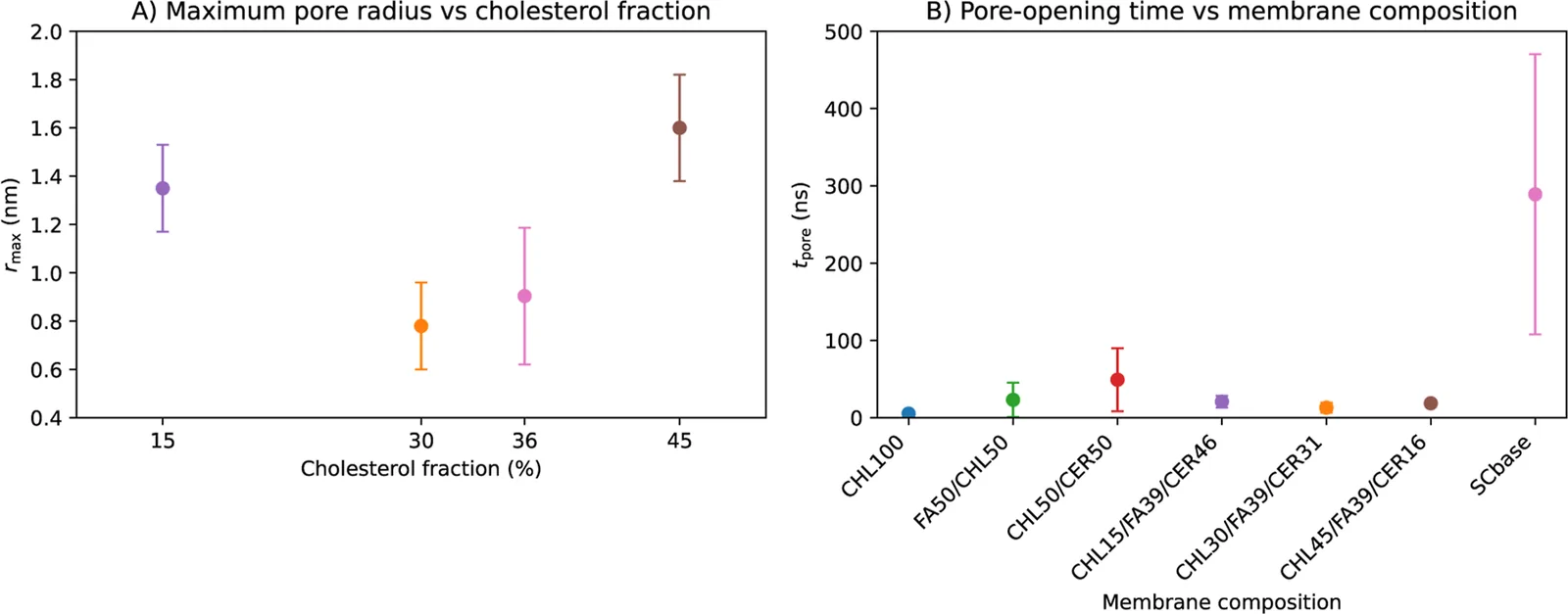
Electric-field-induced pore formation across biomimetic SC lipid membranes (*E*_*z*_ = 1.739 V nm^–1^). (A) Maximum pore radius (*r*_max_) as a function of cholesterol fraction. Individual points correspond to independent replicas, while overlaid symbols indicate mean ± standard deviation when multiple pore-forming events are observed. (B) Mean pore-opening time (*t*_pore_) as a function of membrane composition. Values are reported as mean ± standard deviation over replicas classified as *sustained pore*, reflecting the intrinsic variability of pore nucleation.

Model 2 (CHL100) exhibited rapid pore nucleation in all replicas, with short opening times (*t*_pore_ = 5.37 ± 1.99 ns) and the largest pore sizes (*r*_max_ = 2.92 ± 0.39 nm), indicating a mechanically permissive packing state that stabilizes water defects under electric loading (Figure 4B).

Models 5–9 exhibited pore nucleation on the tens-of-nanoseconds time scale, but with clear differences in postnucleation pore growth. Model 5 (FA50/CHL50) forms comparatively small pores (*r*_max_ = 0.797 ± 0.150 nm), whereas model 6 (CHL50/CER50) exhibits substantially larger defects (*r*_max_ = 2.831 ± 0.315 nm), indicating efficient postnucleation pore expansion. This contrast demonstrates that similar nucleation time scales do not necessarily imply similar pore growth behavior (Figure 4A).

The cholesterol-variation models (models 7–9) exhibit intermediate pore sizes (*r*_max_ ∼ 0.78–1.60 nm) and consistent nucleation across replicas, reinforcing their placement between ceramide-rich resistance and cholesterol-rich permissiveness (Figure 4A,B). In contrast, model 10 (CHL60/FA39/CER01) showed no pore nucleation within the simulated time window, indicating that high cholesterol content embedded within a residual ceramide scaffold restores resistance despite geometric thinning. This comparison highlights that cholesterol alone (model 2) and cholesterol embedded within a biomimetic SC lipid membrane architecture (model 10) correspond to distinct packing regimes with different nucleation barriers.

Model 11 (SCbase) exhibited the longest pore-opening times among the porating compositions, with *t*_pore_ = 289.08 ± 181.16 ns across five independent replicas (Figure 4B), indicating reduced susceptibility relative to other systems under identical field conditions. Despite this delayed response, the resulting defects remained comparatively small (*r*_max_ = 0.904 ± 0.283 nm; Figure 4A), consistent with a mechanically resilient membrane in which pore formation is possible but not strongly favored. The large dispersion in pore-opening times further indicates a strongly nucleation-limited regime.

These results indicate that electroporation in biomimetic SC lipid membranes involves two partially decoupled processes: pore nucleation (Figure 4B) and subsequent pore growth (Figure 4A), each governed by distinct aspects of membrane packing.

To further characterize the progressive loss of membrane integrity under electrical loading, we quantified the area-normalized early water-crossing flux from the onset of the electric field until 5 ns after pore nucleation (Figure 5). Unlike the pore nucleation time, which identifies the onset of a conductive defect, this metric captures the dynamic permeabilization of the membrane during the early stages of electroporation. Binary lipid mixtures revealed a strong dependence on lipid identity (Figure 5A). The FA50/CER50 membrane exhibited virtually no detectable water crossing, whereas replacing ceramides with cholesterol (FA50/CHL50) markedly increased the water-crossing flux. In contrast, the CHL50/CER50 membrane maintained a substantially lower flux, indicating that ceramides play a dominant role in preserving barrier integrity under electrical perturbation. A consistent overall tendency was also observed for the cholesterol-variation series (Figure 5B), in which increasing cholesterol content was generally associated with lower early water-crossing flux. Notably, the physiologically balanced SCbase membrane exhibited an even lower flux than membranes with comparable cholesterol content, indicating that its enhanced resistance cannot be attributed to cholesterol concentration alone, but rather to the cooperative molecular organization arising from the balanced proportions of cholesterol, ceramides, and free fatty acids. Together with the structural descriptors (*D*_HH_, *S*_CD_, tilt angle, and APL), these results support the conclusion that membrane composition mechanically encodes resistance to electric-field-induced pore formation and the accompanying loss of barrier integrity.

**Figure 5:**
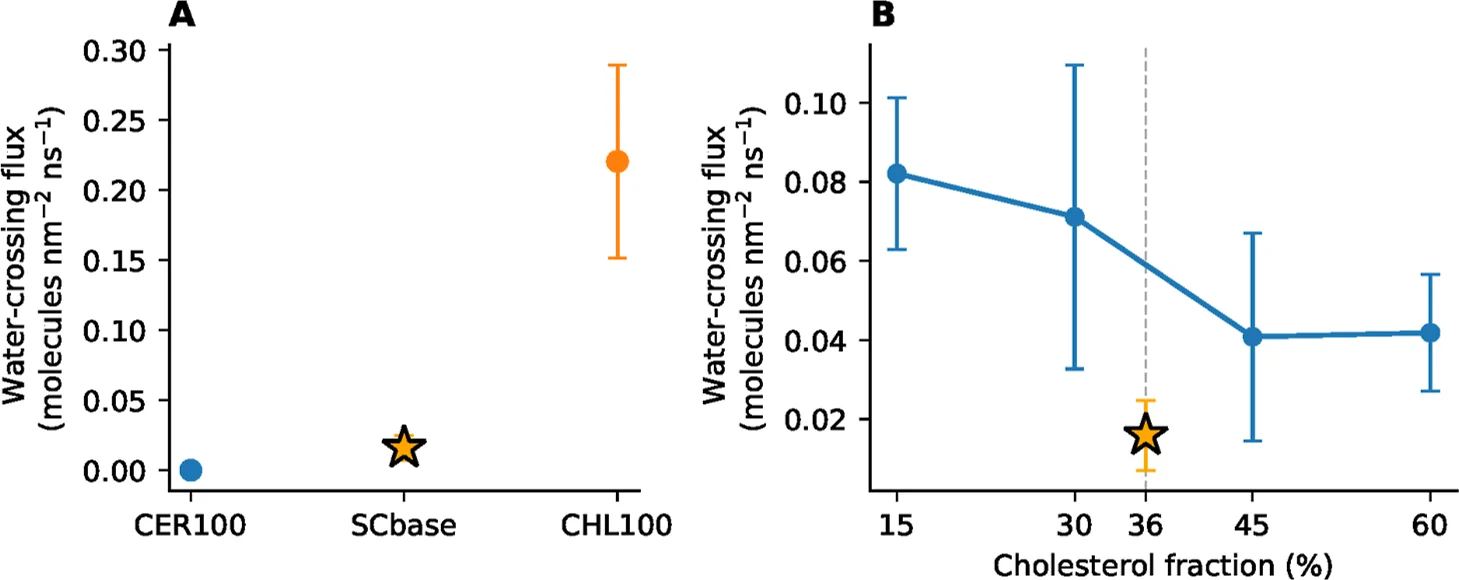
Area-normalized early water-crossing flux as a dynamic descriptor of membrane integrity during electric-field-induced pore formation. (A) Early water-crossing flux for representative binary lipid mixtures (FA50/CER50, FA50/CHL50, and CHL50/CER50), illustrating the influence of lipid identity on membrane integrity under electrical perturbation. (B) Dependence of the early water-crossing flux on cholesterol enrichment for the compositional series containing 15–60 mol % cholesterol. The physiologically balanced SCbase membrane (36 mol % cholesterol) is shown for comparison. The early water-crossing flux was calculated from the onset of the electric field until 5 ns after pore nucleation and normalized by the mean projected membrane area and the corresponding analysis time. Error bars represent the standard deviation obtained from five independent simulations.

### Linking Electroporation Susceptibility to the Mechanical Baseline

The hierarchy of observed pore-opening events defines a composition-dependent susceptibility landscape that reflects the prefield mechanical state. Membranes that resisted poration within the simulated time window (e.g., model 1 and model 10) occupy cohesive packing regimes characterized by preserved interfacial organization and strong ceramide-mediated structural locking. In contrast, compositions that consistently exhibited early pore nucleation and/or formed larger defects (notably model 2 and intermediate models 7–9) reside in mechanically permissive states that promote electric-field-induced stabilization of water defects.

Because APL varies only modestly across biomimetic SC lipid membranes (Table S1), the dominant control axis is vertical and orientational organization rather than global lateral expansion. Electroporation susceptibility is therefore encoded in the equilibrium balance between bilayer thickness, ceramide order, and interfacial locking, rather than dictated by the abundance of any single lipid component. The contrast between model 2 and model 10 further shows that cholesterol does not act as a simple destabilizer: pure cholesterol forms a rapidly porating, defect-stabilizing assembly, whereas high cholesterol embedded within a residual ceramide scaffold restores resistance. Electroporation thus acts as a controlled mechanical probe that reveals latent fragility encoded in lipid packing. Pore nucleation is therefore not solely driven by the external perturbation, but is governed by the underlying free-energy landscape defined by equilibrium membrane organization.

### Bilayer Thickness as a Primary Mechanical Axis of Electroporation Susceptibility

A clear structure–kinetics relationship emerges when observed pore-opening events are mapped onto the equilibrium mechanical baseline. Within biomimetic SC lipid membranes containing ceramides, reduced *D*_HH_ generally accompanies earlier pore nucleation, suggesting that cholesterol-driven thinning shifts the system toward a more permissive vertical packing state under identical electric-field conditions. This trend is most clearly expressed across models 7–10, where *D*_HH_ decreases systematically with increasing cholesterol fraction (Figure 1).

Thickness alone, however, is not sufficient to predict susceptibility. Model 10 is among the thinnest membranes, yet no pore nucleation events were observed within the simulated time window, indicating that the nucleation barrier is jointly controlled by geometric compression and the integrity of the ceramide-mediated interfacial scaffold. We therefore adopt a reduced, table-based mechanical description in which susceptibility is interpreted through four equilibrium parameters extracted from field-free trajectories: *D*_HH_, *S*_CD_^CER^, Tilt_CER_, and APL (Table 2). *D*_HH_ captures the dominant vertical packing axis, *S*_CD_^CER^ and Tilt_CER_ report on ceramide conformational order and orientational locking at the interface, and APL summarizes lateral packing without implying large-scale phase changes.

### Morphological Correlates of Electroporation: Snapshot-Level Structural Evidence

To connect pore nucleation events (*t*_pore_) and pore-size descriptors (*r*_max_, *r*_final_) with physical membrane failure modes, we inspected representative snapshots of selected models. Figures 6–8 provide morphology-level evidence consistent with the hierarchy derived from Table 3.

**Figure 6:**
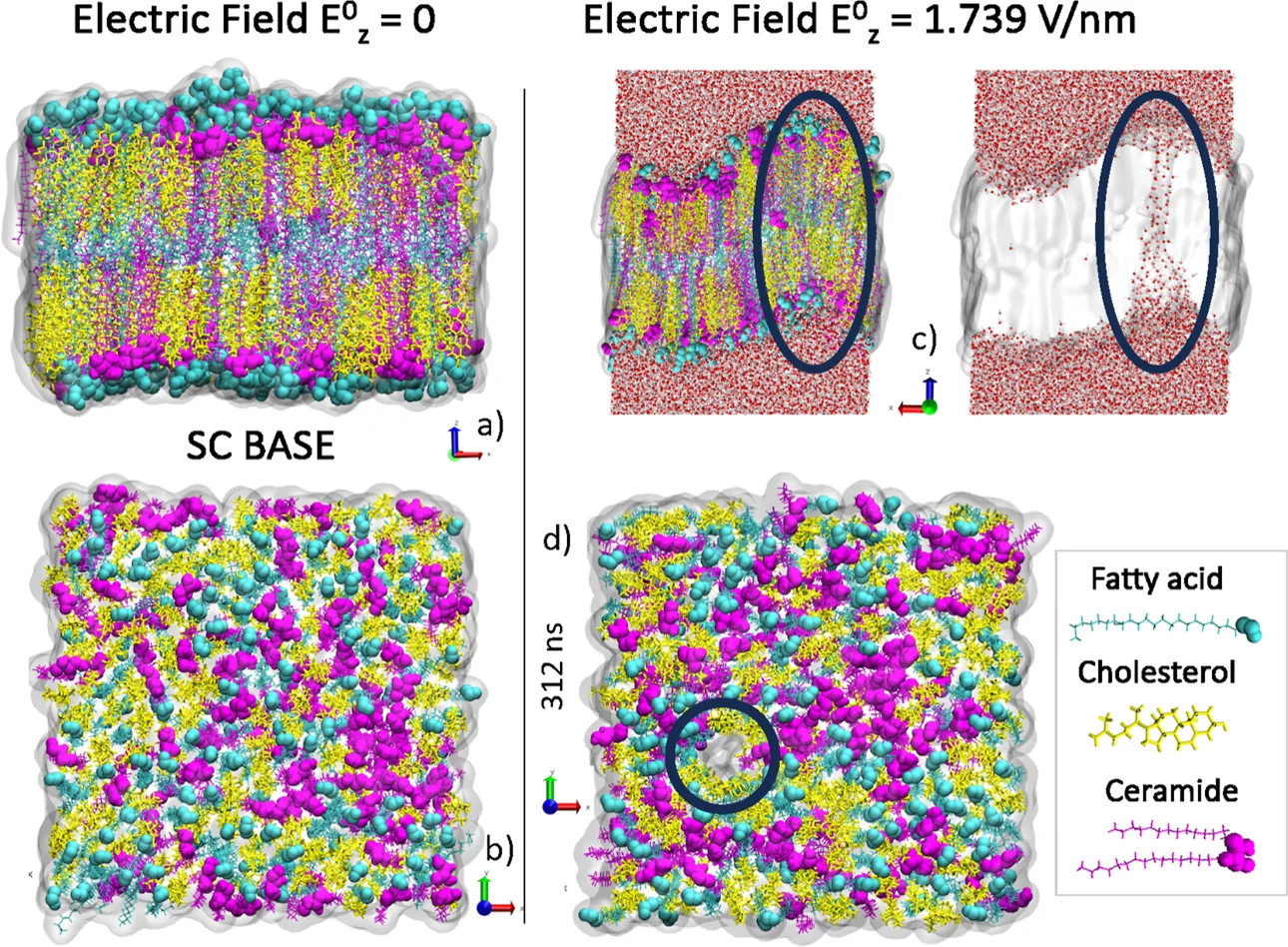
Structural baseline and delayed pore nucleation in model 11 (SCbase). (a) Front view under control conditions (water/ions removed for clarity). (b) Top view of the same configuration. (c) Snapshot at pore nucleation under *E*_*z*_ = 1.739 V nm^–1^: full system (left) and semitransparent rendering highlighting the hydrated defect (right). (d) Top view highlighting the localized conductive pathway.

Model 11 remains well organized under field-free conditions and nucleates a conductive defect only at late times under *E*_*z*_ = 1.739 V nm^–1^. The resulting pore remains comparatively small, consistent with a nucleation-limited response rather than rapid postnucleation expansion (Figure 6).

Similarly, model 4 remains intact under the applied field, consistent with the *no wetting* classification across replicas. Snapshot inspection confirms the absence of a continuous transmembrane water channel (Figure 7), supporting a mechanically cohesive and nucleation-resistant packing state.

**Figure 7:**
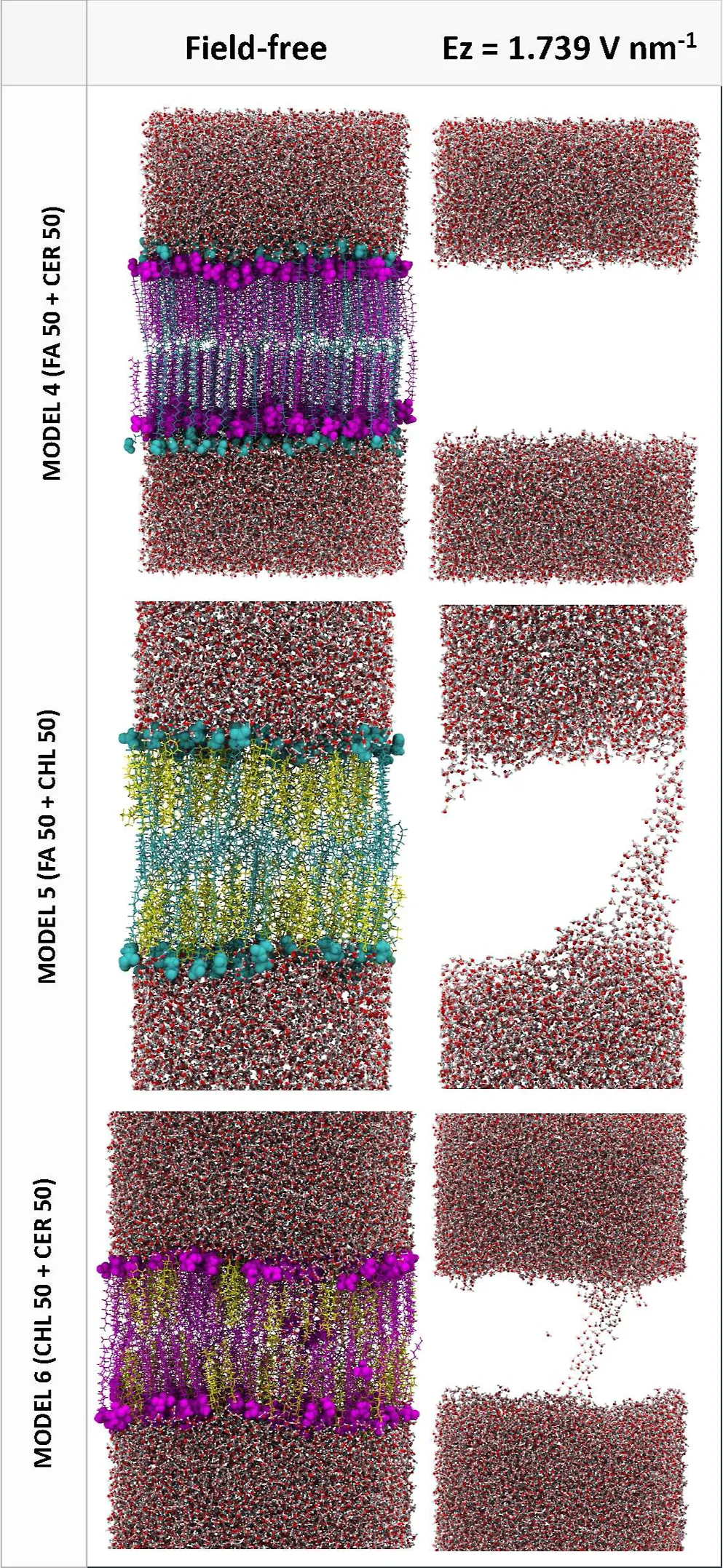
Morphological response of binary biomimetic SC lipid membranes under electric loading. Representative snapshots of models 4–6. Left: field-free configurations; right: under *E*_*z*_ = 1.739 V nm^–1^. Model 4 remains intact with no continuous hydrated defect (*no wetting*). Model 5 exhibits defect nucleation and formation of a conductive aqueous pathway. Model 6 shows pore formation followed by substantial pore expansion, consistent with its large *r*_max_ values. Water is shown only under field for clarity.

In contrast, model 5 shows electric-field-induced defect nucleation and formation of a continuous water channel, consistent with the *sustained pore* classification. This behavior illustrates an intermediate mechanical regime between ceramide-rich resistance and cholesterol-rich permissiveness (Figure 7).

At the opposite extreme, model 2 nucleates pores rapidly and forms large conductive channels, consistent with the shortest observed *t*_pore_ values and largest *r*_max_ values among all systems (Figure 8). This behavior reflects a mechanically permissive packing state that facilitates stabilization and growth of water defects under sustained electric fields.

**Figure 8:**
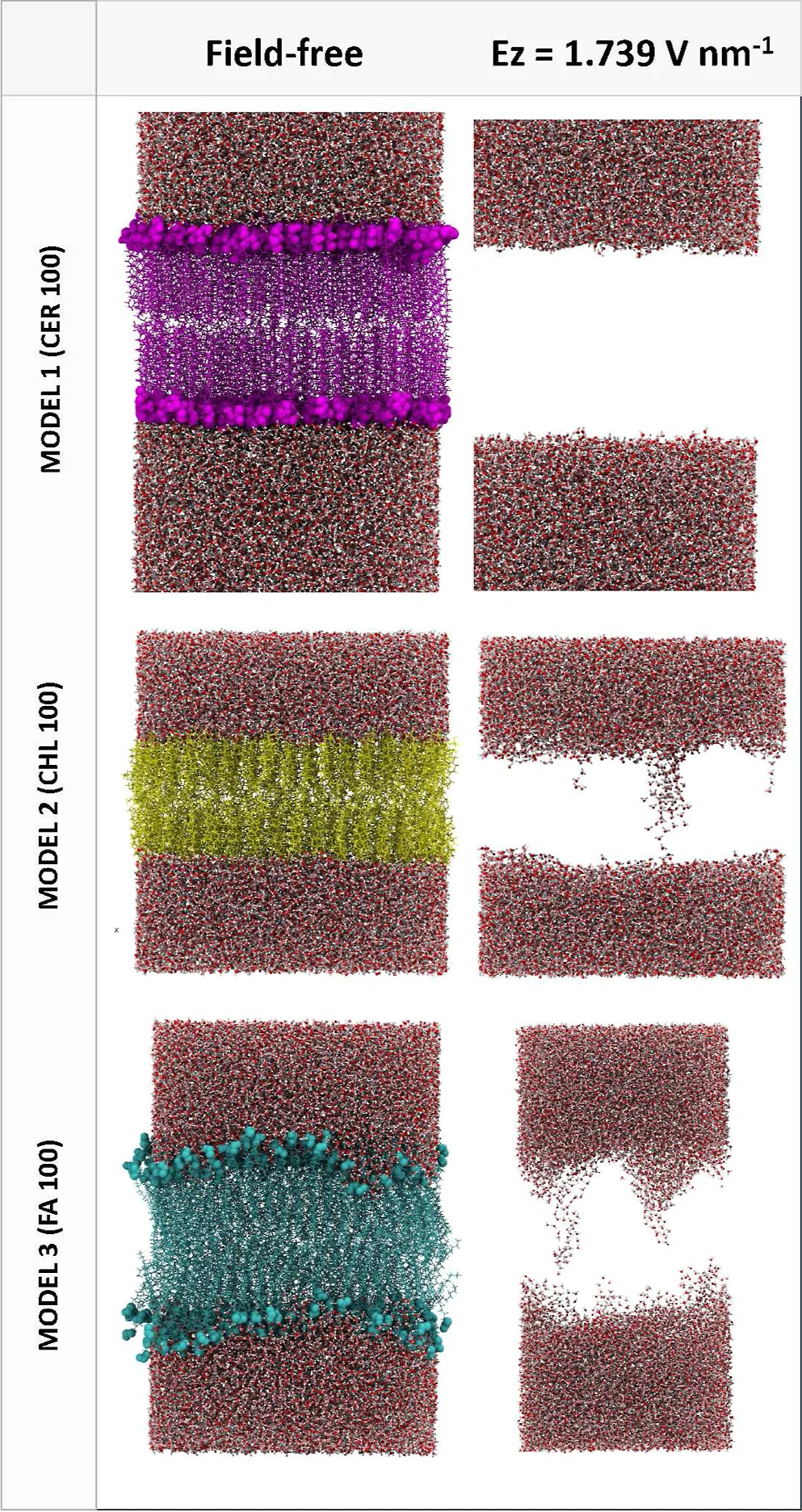
Extreme packing states and failure modes of biomimetic SC lipid membranes under electric loading. Representative snapshots of models 1–3. Left: field-free configurations; right: under *E*_*z*_ = 1.739 V nm^–1^. Model 1 preserves structural integrity with no hydrated defect. Model 2 exhibits rapid pore nucleation and large conductive pathways. Model 3 shows intrinsic structural instability and abnormal hydration under control conditions, precluding clear separation between equilibrium and field-induced effects. Water is shown only under field for clarity.

Model 3 exhibits abnormal core hydration and reduced interfacial cohesion even under control conditions, precluding unambiguous attribution of defect formation to the applied field. This intrinsic instability prevents a clear separation between equilibrium fragility and field-induced poration, and the system was therefore excluded from quantitative electroporation comparisons (Figure 8).

Consistent with the structural baseline, model 1 maintains integrity and shows no continuous transmembrane water channel under the applied field, reflecting its highly cohesive and interfacially locked packing state (Figure 8).

### Compositional Packing, Defect Nucleation, and Mechanical Fragility

Electric-field-induced pore nucleation provides a controlled framework to interrogate how equilibrium packing determines membrane failure. Rather than interpreting electroporation solely as an external perturbation, it can be viewed as a stress test that exposes latent structural fragility encoded in lipid organization.

Previous experimental and computational studies of stratum corneum model membranes have demonstrated that ceramides act as primary interfacial scaffolds, enforcing lamellar cohesion and suppressing packing defects.^35–37^ In contrast, cholesterol modulates vertical packing and leaflet organization without necessarily inducing large lateral expansion. Our results align with this picture: across biomimetic SC lipid membranes, APL remains nearly conserved while *D*_HH_ decreases systematically with cholesterol enrichment, indicating that sterol-driven reorganization operates primarily along the vertical/orientational axis.

Electroporation kinetics reveal how these equilibrium packing differences translate into defect nucleation barriers. Theoretical treatments and molecular simulations have shown that pore formation energy scales with membrane thickness and local packing order.^38,39^ In this framework, reduced thickness lowers the dielectric and mechanical cost of water penetration, but nucleation ultimately depends on the presence of local packing imperfections rather than global disorder.

This distinction explains the contrast between model 2 and model 10. Pure cholesterol forms a rapidly porating assembly, consistent with studies linking sterol-rich membranes to enhanced local defect stabilization.^40^ However, when cholesterol is embedded within even a minimal ceramide scaffold, interfacial locking is restored, and pore nucleation is suppressed despite geometric thinning. Thus, electroporation susceptibility is governed by integrated packing states rather than cholesterol fraction alone.

These results reveal that pore nucleation kinetics and pore growth are partially decoupled processes. While models 5 and 6 exhibit comparable nucleation time scales, they differ markedly in pore size, indicating that postnucleation expansion is strongly dependent on the underlying mechanical packing state.

Importantly, the SCbase composition (model 11) occupies a mechanically optimized regime in which moderate thinning coexists with preserved interfacial cohesion. This balance yields delayed pore nucleation under identical electrical loading, suggesting that native-like compositional complexity minimizes defect probability while retaining structural adaptability.

Together, these findings support a defect-mediated nucleation model in which bilayer thickness defines the dominant mechanical axis, while ceramide order and orientational locking modulate the effective free energy barrier for water-defect stabilization. In this sense, electroporation susceptibility is not dictated by lipid identity alone, but emerges from the integrated equilibrium packing state of the membrane.

## Conclusions

We establish a composition–structure–mechanics–electroporation relationship for biomimetic stratum corneum lipid membranes under constant electric loading. By integrating equilibrium structural descriptors (*D*_HH_, *S*_CD_, tilt, and APL) with pore nucleation metrics (*t*_pore_ and *r*_max_), we show that susceptibility to electric-field-induced pore formation is largely encoded in the intrinsic mechanical baseline of the membrane rather than dictated by any single lipid component.

Our compositional design spans a broad range of lipid balances representing both physiologically balanced and perturbed membrane states. Within this framework, ceramide-rich assemblies define cohesive, nucleation-resistant regimes characterized by strong interfacial locking and core dehydration, whereas cholesterol modulates the mechanical baseline primarily through vertical and orientational reorganization, reducing bilayer thickness without substantially altering lateral packing.

The resulting electroporation response, together with the time-resolved water transport analysis, reflects these compositional and mechanical differences. Pure cholesterol membranes exhibit rapid pore nucleation and form large conductive defects, whereas the physiologically balanced SCbase composition exhibits delayed pore nucleation kinetics (*t*_pore_ = 289.08 ± 181.16 ns) and comparatively small pore sizes (*r*_max_ = 0.904 ± 0.283 nm), consistent with a mechanically resilient yet not pore-immune regime. Importantly, cholesterol embedded within a preserved ceramide scaffold restores resistance despite geometric thinning, demonstrating that nucleation barriers emerge from integrated packing states rather than lipid fraction alone.

These results position electroporation as a quantitative mechanical probe of compositional fragility in SC lipid assemblies. By revealing how lipid balance controls both defect nucleation and the early loss of water-barrier integrity under electrical polarization, this work establishes a predictive framework linking molecular organization to barrier resilience.

Importantly, this framework provides a mechanistic basis for interpreting how compositional alterations—such as those observed in skin disorders—may influence electrically assisted transport across the SC. These insights have direct implications for transdermal delivery, skin barrier dysfunction, electroporation-based permeabilization strategies, and the rational design of lipid-based systems for controlled permeability modulation.

## Supplementary Material



## Notes

Equilibrium structural descriptors and density profiles for the membrane models; per-replica pore-nucleation times, pore-size descriptors, and area-normalized water-crossing fluxes; representative time-resolved water-integrity and cumulative transmembrane water-crossing analyses; and temperature stability during electric-field simulations (PDF)
