# Supplementary material for: From Lipid Composition to Electroporation: Mechanical Encoding of Barrier Resilience in Stratum Corneum Membranes
Source: ACS Omega. 2026 Sep 1;11(36):54029–40. doi: 10.1021/acsomega.6c04148 (PMC13584771; doi:10.1021/acsomega.6c04148)
Supplement: Supplementary file 1 [file ao6c04148_si_001.pdf]

Supporting Information: Supporting Information for "From Lipid Composition to Electroporation: Mechanical Encoding of Barrier Resilience in Stratum Corneum Membranes"

Neila Cristina Fonseca Machado* , Nicolás A. García , Nuria Anguita-Ortiz , Lorena Ruano , Juan J. Nogueira , and Herculano da Silva Martinho*

E-mail:
neilamachado@gmail.com/neila.machado@ufabc.edu.br/herculano.martinho@ufabc.edu.br

This Supporting Information provides the complete structural and kinetic datasets supporting the mechanistic framework developed in the main text. It includes (i) integrated equilibrium descriptors for all control membranes, (ii) per-replica electroporation outcomes under a constant electric field, and (iii) density-resolved structural analyses.

The reduced descriptor set in the main text (Table 1) is extracted from the full structural dataset reported here, isolating the dominant variables governing pore nucleation susceptibility.

Table S1: **Integrated equilibrium structural descriptors for all control membranes.** Values were computed from field-free trajectories using the final 100 ns. D_{HH} was obtained from headgroup density maxima. S_{CD} values correspond to chain-averaged deuterium order parameters. $S_{\mathrm{axis}}^{\mathrm{CHL}}$ is defined along the C3→C17 molecular axis. Tilt angles (degrees) are defined relative to the membrane normal. APL values are reported as mean ± standard deviation over the final 100 ns. Model numbering follows the nomenclature introduced in the main text.

Model	D_{HH} (Å)	S_{CD}^{CER}	S_{CD}^{FA}	$S_{\mathrm{axis}}^{\mathrm{CHL}}$	$\mathrm{Tilt}_{\mathrm{CER}}$	$\mathrm{Tilt}_{\mathrm{FA}}$	$\mathrm{Tilt}_{\mathrm{CHL}}$	APL (nm^2)
Model 1 (CER100)	41.5	−0.407	–	–	13.9	–	–	0.383 ± 0.001
Model 2 (CHL100)	33.5	–	–	0.925	–	–	11.3	0.381 ± 0.001
Model 3 (FA100)	60.5	–	−0.103	–	–	16.2	–	0.215 ± 0.001
Model 4 (FA50/CER50)	43.5	−0.409	−0.102	–	14.2	7.0	–	0.286 ± 0.001
Model 5 (FA50/CHL50)	41.5	–	−0.082	0.947	–	10.8	9.5	0.290 ± 0.001
Model 6 (CHL50/CER50)	37.0	−0.366	–	0.950	16.7	–	9.2	0.385 ± 0.001
Model 7	42.0	−0.402	−0.085	0.964	14.2	7.9	9.0	0.307 ± 0.001
Model 8	40.5	−0.372	−0.085	0.943	16.9	8.4	9.6	0.308 ± 0.001
Model 9	37.5	−0.366	−0.077	0.953	15.8	9.1	10.2	0.309 ± 0.001
Model 10	34.5	−0.371	−0.062	0.944	19.4	9.9	10.8	0.307 ± 0.001
Model 11 (SCbase)	39.0	−0.366	−0.080	0.951	18.0	8.8	9.9	0.309 ± 0.001

Table S2: **Per-replica electroporation outcomes under a constant electric field ($E_z = 1.739$ V nm^{-1}).** For each model and independent replica, we report pore classification, pore-opening time t_{pore}, pore size descriptors ($r_{\max}$ and r_{final}), and the area-normalized early water-crossing flux (J_{water}). Pore formation was identified based on a hydration persistence criterion. J_{water} was calculated by normalizing the cumulative number of water-crossing events by the mean projected membrane area and the corresponding analysis time. For porating trajectories, the analysis was performed from the onset of the electric field until 5 ns after pore nucleation; for non-porating trajectories, the available analyzed trajectory window was used. Model 3 (FA100) was not included in the quantitative water-crossing analysis because its intrinsic structural instability and abnormal core hydration under field-free conditions preclude a clear separation between equilibrium fragility and electric-field-induced transport. Entries marked "–" indicate absence of pore formation within the simulated time window; "n.a." indicates not applicable.

Model	E_z (V nm^{-1})	Seed	Classification	t_{pore} (ns)	$r_{\max}$ (nm)	r_{final} (nm)	J_{water} (molecules nm^{-2} ns^{-1})
Model 1	1.739	1	no wetting	–	–	–	0.000
Model 1	1.739	2	no wetting	–	–	–	0.000
Model 1	1.739	3	no wetting	–	–	–	0.000
Model 2	1.739	1	sustained pore	3.20	3.04	2.93	0.143
Model 2	1.739	2	sustained pore	5.80	2.48	2.42	0.275
Model 2	1.739	3	sustained pore	7.10	3.23	3.17	0.243
Model 3	1.739	1	excluded	–	–	–	n.a.
Model 3	1.739	2	excluded	–	–	–	n.a.
Model 3	1.739	3	excluded	–	–	–	n.a.
Model 4	1.739	1	no wetting	–	–	–	0.000
Model 4	1.739	2	no wetting	–	–	–	0.000
Model 4	1.739	3	no wetting	–	–	–	0.000
Model 5	1.739	1	sustained pore	12.2	0.9271	0.9271	0.059
Model 5	1.739	2	sustained pore	48.6	0.6333	0.5352	0.061
Model 5	1.739	3	sustained pore	8.8	0.8292	0.8292	0.036
Model 6	1.739	1	sustained pore	57.8	3.1935	3.1890	0.011
Model 6	1.739	2	sustained pore	37.1	2.6655	2.6655	0.007
Model 6	1.739	3	sustained pore	94.7	2.6330	2.6330	0.015
Model 7	1.739	1	sustained pore	9.10	1.47	1.46	0.091
Model 7	1.739	2	sustained pore	23.80	1.57	1.57	0.103
Model 7	1.739	3	sustained pore	22.90	1.38	1.38	0.070
Model 7	1.739	4	sustained pore	18.90	1.26	1.14	0.055
Model 7	1.739	5	sustained pore	29.90	1.10	0.97	0.090
Model 8	1.739	1	sustained pore	9.00	0.81	0.81	0.102
Model 8	1.739	2	sustained pore	5.10	0.97	0.97	0.120
Model 8	1.739	3	sustained pore	14.50	0.61	0.59	0.060
Model 8	1.739	4	sustained pore	22.40	0.93	0.93	0.032
Model 8	1.739	5	sustained pore	14.50	0.59	0.56	0.041
Model 9	1.739	1	sustained pore	22.00	1.38	1.38	0.019
Model 9	1.739	2	sustained pore	16.60	1.59	1.59	0.078
Model 9	1.739	3	sustained pore	23.50	1.53	1.53	0.018
Model 9	1.739	4	sustained pore	15.20	1.52	1.52	0.058
Model 9	1.739	5	sustained pore	16.40	1.97	1.97	0.031
Model 10	1.739	1	no wetting	–	–	–	0.051
Model 10	1.739	2	no wetting	–	–	–	0.032
Model 10	1.739	3	no wetting	–	–	–	0.033
Model 10	1.739	4	no wetting	–	–	–	0.030
Model 10	1.739	5	no wetting	–	–	–	0.064
Model 11	1.739	1	sustained pore	312.7	0.6555	0.5614	0.019
Model 11	1.739	2	sustained pore	174.1	1.4060	1.4060	0.007
Model 11	1.739	3	sustained pore	221.1	0.6770	0.5352	0.019
Model 11	1.739	4	sustained pore	606.3	1.0434	1.0434	0.007
Model 11	1.739	5	sustained pore	131.2	0.7378	0.7378	0.028

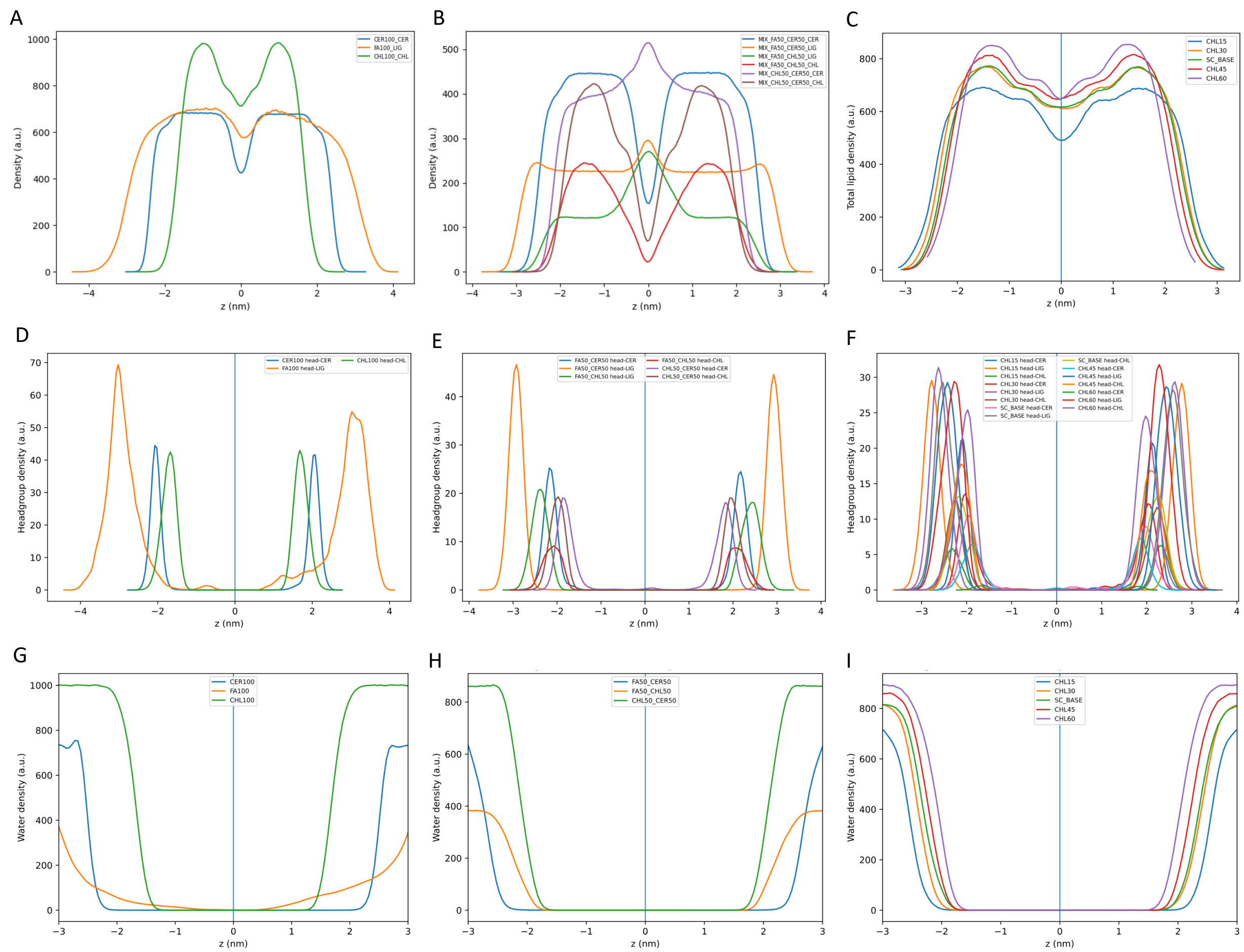

Figure S1: **Density-resolved structural baseline of biomimetic SC lipid membranes.** Top row: total and component lipid mass density profiles for (A) extreme compositions, (B) binary mixtures, and (C) biomimetic SC lipid membrane compositions (Models 7–10 and SCbase), illustrating cholesterol-dependent bilayer thinning and variation in global membrane envelope. Middle row: headgroup-resolved density profiles for (D–F), revealing the layered SC architecture, with ceramide-enriched interfacial regions, fatty-acid contributions to the hydrophobic core, and cholesterol positioned between headgroups and the membrane interior. Bottom row: water density profiles for (G–I), reporting core dehydration across compositions and highlighting latent hydration signatures under field-free conditions. These density profiles provide the structural basis for the equilibrium descriptors used to define the mechanical baseline in the main text.

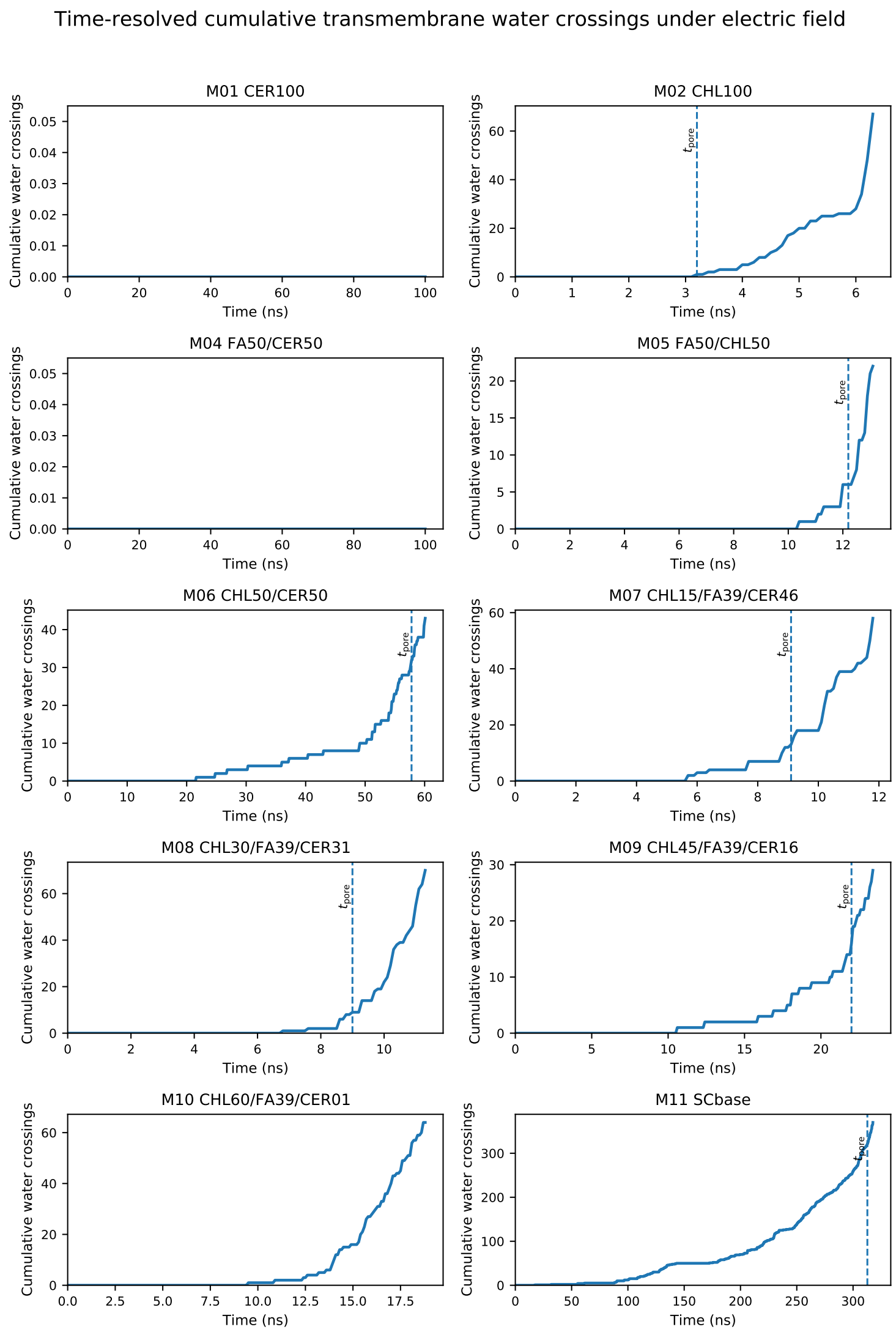

Figure S2: **Time-resolved cumulative transmembrane water crossings under a constant electric field.** Representative cumulative transmembrane water-crossing events as a function of simulation time for the Seed 1 trajectory of each membrane composition analyzed under a constant electric field ($E_z = 1.739$ V nm^{-1}). Each curve corresponds to the cumulative number of complete water molecules that traversed the membrane from one bulk aqueous compartment to the opposite compartment according to the crossing criterion described in the Methods. Vertical dashed lines indicate the pore nucleation time (t_{pore}) for porating trajectories. Membranes that did not undergo pore formation during the analyzed time window are shown without a t_{pore} marker. These trajectories illustrate the temporal evolution of water transport and demonstrate that, depending on membrane composition, water permeation may progressively increase before the onset of pore nucleation and accelerate after pore formation. Model 3 (FA100) was omitted because this membrane was excluded from the structural analyses owing to its intrinsic instability under the simulation conditions.

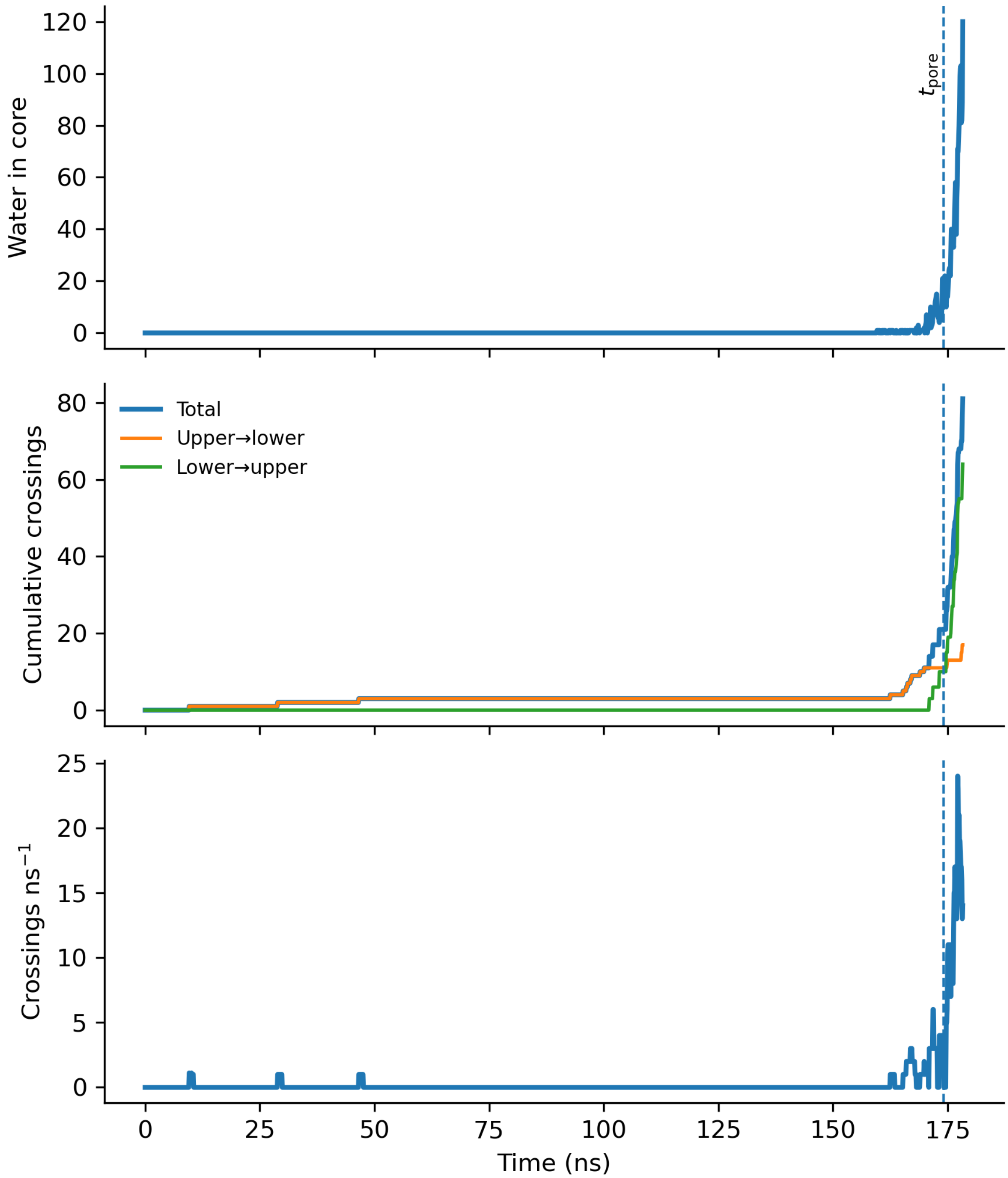

Figure S3: **Representative time-resolved water-integrity analysis for the SCbase membrane.** Example trajectory for Model 11 (SCbase), seed 2, under a constant electric field ($E_z = 1.739$ V nm^{-1}). The upper panel shows the number of water molecules within the membrane core, the middle panel shows the cumulative number of complete transmembrane water crossing events, and the lower panel shows the instantaneous water-crossing rate. The vertical dashed line marks the pore-opening time, $t_{\text{pore}} = 174.1$ ns. For this trajectory, 81 water-crossing events were detected over the analyzed window, yielding an area-normalized early water-crossing flux of $J_{\text{water}} = 0.007$ molecules nm^{-2} ns^{-1}.

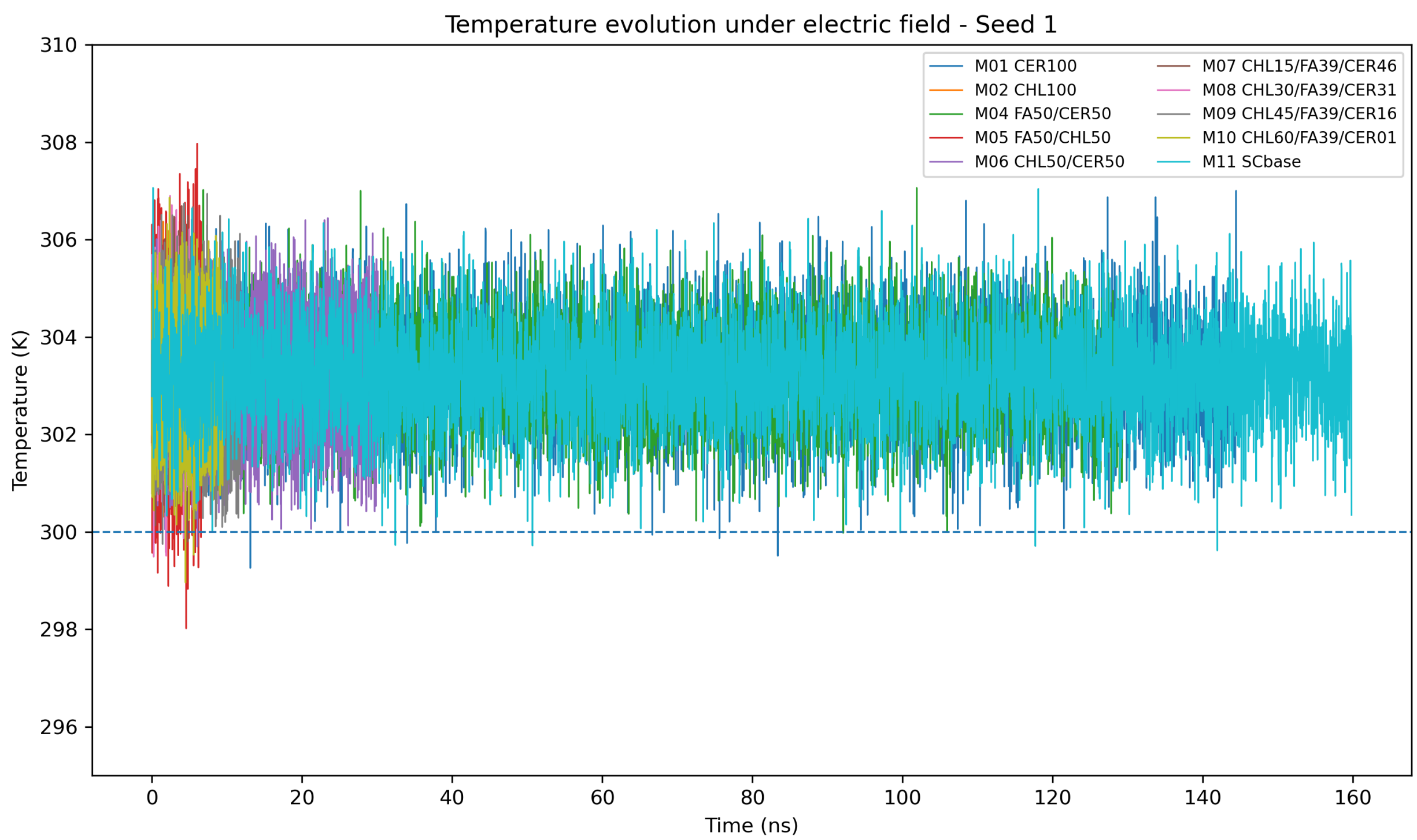

Figure S4: **Temperature stability during constant-electric-field simulations.** Time evolution of the instantaneous temperature for the Seed 1 trajectories of each membrane composition simulated under a constant external electric field ($E_z = 1.739\ \mathrm{V\,nm^{-1}}$). All simulations were performed using a Langevin thermostat with a target temperature of 300 K. The temperature remained close to the target value throughout the simulations, despite the different simulation lengths associated with pore formation, with no evidence of systematic temperature drift or progressive field-induced heating. The FA100 membrane was omitted because this composition was excluded from the structural analyses owing to its intrinsic instability, as described in the main text.
